# Using off-gas for insights through online monitoring of ethanol and baker’s yeast volatilome using SESI-Orbitrap MS

**DOI:** 10.1038/s41598-022-16554-z

**Published:** 2022-07-21

**Authors:** Hendrik G. Mengers, Martin Zimmermann, Lars M. Blank

**Affiliations:** Institute of Applied Microbiology - iAMB, Aachener Biology and Biotechnology - ABBt, RWTH Aachen University, Aachen, Germany

**Keywords:** Mass spectrometry, Metabolomics

## Abstract

Volatile organic compounds play an essential role in every domain of life, with diverse functions. In this study, we use novel secondary electrospray ionisation high-resolution Orbitrap mass spectrometry (SESI-Orbitrap MS) to monitor the complete yeast volatilome every 2.3 s. Over 200 metabolites were identified during growth in shake flasks and bioreactor cultivations, all with their unique intensity profile. Special attention was paid to ethanol as biotech largest product and to acetaldehyde as an example of a low-abundance but highly-volatile metabolite. While HPLC and Orbitrap measurements show a high agreement for ethanol, acetaldehyde could be measured five hours earlier in the SESI-Orbitrap MS. Volatilome shifts are visible, e.g. after glucose depletion, fatty acids are converted to ethyl esters in a detoxification mechanism after stopped fatty acid biosynthesis. This work showcases the SESI-Orbitrap MS system for tracking microbial physiology without the need for sampling and for time-resolved discoveries during metabolic transitions.

## Introduction

The sensation of “smell” caused by volatile molecules is not restricted to animals but is native to all domains of life. The sum of all such molecules is named the volatilome. The effectors are most often volatile organic compounds (VOCs), hydrocarbons with low molecular masses and high vapour pressures^1^. Purposefully produced VOCs play an important role in inter- and intraspecies communication. A comprehensive review of their functions and chemical properties is given by Weisskopf et al.^2^. For humans, the VOC contributions to taste and smell are particularly interesting. In simplest terms, this means distinguishing quality food from rotten equivalents^3^.

The yeast *Saccharomyces cerevisiae* was used for most of human civilization and is at least timely correlated to humans settling down^4^. It has been used for the production of bread, beer, and wine for at least five thousand years^5^. In the modern world, *S. cerevisiae* is the best-studied microbial eukaryote and one of the major industrial micoorganisms^6^. The main laboratory and reference strain of *S. cerevisiae* is S288C^7^, which was also the first sequenced yeast. The annotated genome now consists of over 6000 protein-coding genes^8^. Further, the genes, metabolites, and metabolic pathways are easily accessible thanks to community efforts like the *Saccharomyces* genome database^9^ and the genome-scale metabolic model Yeast8^10^.

Although yeast’s genome and metabolic pathways are intensely studied, the volatilome is comparatively sparsely explored. Ebert et al. reviewed the origin of yeast volatiles and their detection methods comprehensively^3^. VOCs in yeasts are derived from primary as well as from secondary metabolic pathways and include hydrocarbons, heterocycles, aldehydes, ketones, alcohols, thiols, and many more classes of molecules^3^. But in addition to the described pathways, VOCs can also arise from non-catalysed reactions or reactions catalysed by promiscuous enzymes^11,12^. Volatile metabolites from *S. cerevisiae* are of particular interest in the food and flavour industries, as they cause characteristic smells or off-flavours^13^. Despite the direct smell of these metabolites, they can also give insights into the cell’s metabolic status. Consequently, monitoring the volatilome can allow for a tighter cultivation control and thus improve biotechnological fermentations. Gas chromatography, in combination with solid phase microextraction (SMPE) fibres, are the gold standard for measuring volatile substances and with that for volatilome analysis^14–16^. The challenge with these techniques is sampling. On the one hand, sampling potentially disturbs the biological process, and sampling via SPME is laborious. On the other hand, sampling can produce artefacts. This could either mean introducing contaminants from the sampling equipment (e.g., plastic bags) but also enriching or excluding substances based on their affinity to SPME fibres^17^. Therefore, novel analytical methods are of rising interest. Ambient pressure ionisation (API) mass spectrometry offers the possibility to measure gas-phase samples without distorting the volatilome by high temperatures or low pressures. A conclusive review about of present state of API-MS was published by Rankin-Turner and Heaney^18^.

Alternatively, a secondary electrospray ionisation (SESI) unit can be coupled to a high-resolution Orbitrap mass spectrometer (SESI-Orbitrap MS). In the SESI, an electrospray is generated through which the analyte gas stream is passed. The charged water clusters collide with the gas-phase metabolites and transfer their charge, although this mechanism is not fully elucidated^19^. With this very soft ionisation technique, minimal fragment formation is ensured^20^. Furthermore, using a high-resolution MS enables to bypass the separation step, necessary in GC–MS. This allows for online monitoring with the scan speed of the MS unit, in this case, over 3 Hz. This speed contrasts with a study published by Khomenko et al. using proton transfer time-of-flight (PTR-ToF) MS to analyse the yeast volatilome, where automated headspace measurements were possible only every 4 h^21^, or a study by Link et al., where the microbial culture was analysed by direct injection into an MS^22^. Albeit, without the information from analyte-specific retention times, unknown compounds can only be identified up to their molecular formula. SESI-Orbitrap MS was used in previous studies on the volatilome of plants^23^, humans^24,25^, and also *S*. *cerevisiae*^26^. The latter study investigated the immediate response in ethanol production after a glucose pulse. Through ^13^C glucose experiments, they could show differences in the volatilome of different mutant strains and the wild-type strain focusing on fatty acid ethyl esters.

Here, in this study, the volatilome of growing yeast cultures from lag-phase to stagnation in shake flasks and a reactor was monitored using SESI-Orbitrap MS. With HPLC and GC measurements as a comparison, the usability of this system for tracking ethanol and acetaldehyde online in real-time was demonstrated. Further, metabolic shifts were reflected in changes in the volatilome, measured over 16,0000 times.

## Results and discussion

### Using SESI-Orbitrap MS for gas-phase ethanol measurement

**Figure 1:**
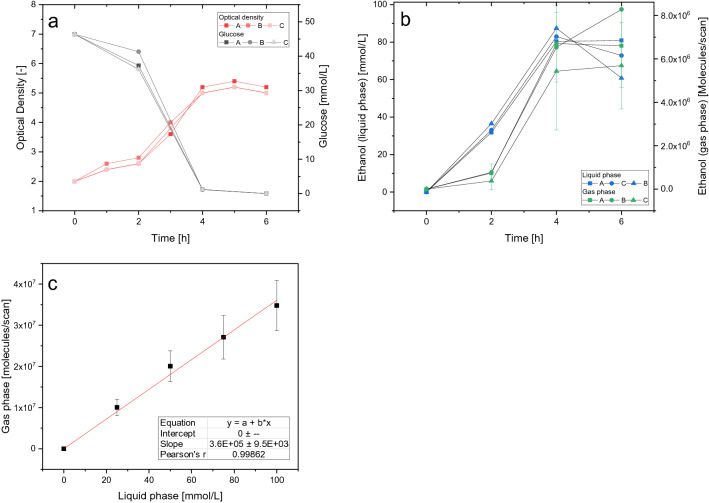
Optical density and glucose concentration of the yeast in shake flasks (**a**). Concentration of ethanol as measured by HPLC (liquid phase) and as intensity SESI-Orbitrap MS (gas-phase), presented are means and SD for n = 3 technical replicates (**b**). Calibration curve of ethanol in the gas phase above an aqueous ethanol solution with 0–100 mM at 30 °C, presented are means and SD for n = 3 technical replicates (**c**).

A preculture of *S. cerevisiae* S288C in Verduyn minimal medium was split into three separate shake flasks with an OD of 2 each. In addition to one sample with uninoculated medium, the flasks were probed every two hours for a total duration of six hours to collect samples for OD, HPLC, and volatilome measurement. The SESI-Orbitrap MS measurements were additionally performed as technical triplicates. The volatilome analysis was performed by placing the shake flask under the extended intake line of the machine (Setup Supplementary Fig. 1.1). The growth of the yeast, as well as the glucose concentration, is shown in Fig. 1a. The cells grew steadily using glucose as the sole carbon and energy source until its depletion shortly after the 4 h measurement point, after which growth stopped. Overall, the biological triplicates are in good agreement with each other.

Ethanol is of particular interest because it is not only biotechnologies volumetrically largest product^27^ but also an important indicator of the metabolic status, e.g., through the Crabtree effect in *S. cerevisiae*, where ethanol is produced even in aerobic conditions if a glucose threshold of approximately 0.1 g L^−1^ is reached^28^. Although with its nominal mass of 46 Da, it is slightly too small to be measured by the SESI-Orbitrap MS-system, with its detection range of 50–500 *m/z*. The ethanol dimer has an [M + H]^+^ of 93.0910 *m/z* and is therefore detectable as a so-called proton-bound dimer^26,29^. With this exact mass, the only possible molecular formula within the 3 ppm uncertainty interval of the machine is C_4_H_12_O_2_. This cannot be an actualmolecule, as the unsaturation would be − 1, which would need a pentavalent carbon. Therefore, a feature with this exact mass can be identified as ethanol (dimer). A calibration curve spanning 0–100 mM ethanol in distilled water with technical triplicates shows a clear correlation between ethanol concentrations in the liquid phase and measured gas phase using SESI-Orbitrap MS with a factor of 3.6 × 10^5^ (Fig. 1c). But this correlation is heavily matrix-dependent, and growing yeast cultures present a changing matrix as discussed later. During the cultivation in shake flasks, the ethanol concentrations in the supernatant were measured using HPLC–UV/RI, whilst the gas phase above the shake flasks was analysed using SESI-Orbitrap MS. Overall, the concentrations measured by HPLC and SESI-Orbitrap MS are in good agreement, although low concentrations are slightly undervalued (Fig. 1b). This is not an artefact from the gas phase measurement, because the ethanol calibration curve did not show this behaviour, but a result of the changing composition of the yeast culture broth.

### Measuring the yeast volatilome from a shake flask culture

**Figure 2:**
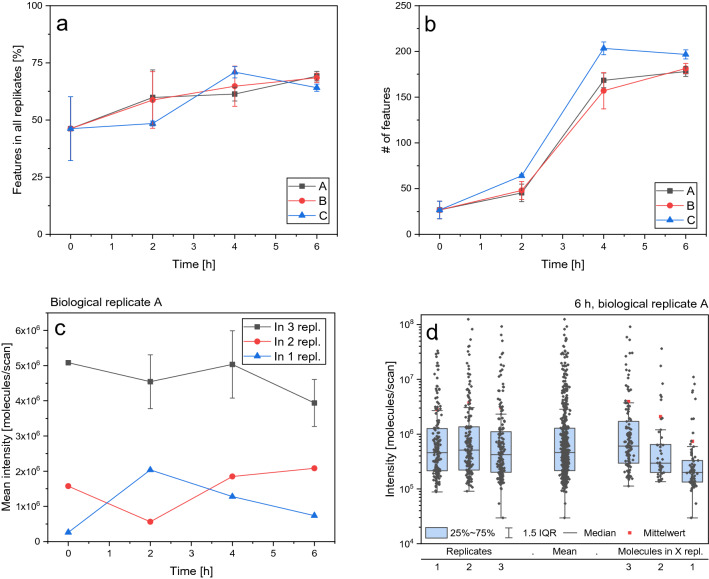
Number of identified volatiles over a growing yeast culture in shake flasks over time, presented are means and SD for n = 3 technical replicates (**a**) Percentage of features that are present in all replicates over time (**b**). Mean intensity of features present in one, two, or all three triplicate runs for shake flask A, error bars just for those n = 3 (**c**). Intensity on a logarithmic scale of every feature in shake flask A at 6 h (left), grouped whether the features are present in one, two, or all three replicate runs (right) (**d**).

Around 25 volatile features could be identified in uninoculated minimal medium (Fig. 2b), which are contaminations gathered throughout the setup of the experiments, as the used salts and vitamins have very low vapour pressures. The number of identified volatiles per measurement increased over time. 50 molecular species were found 2 h after inoculation, whilst 200 could be identified after 6 h. This is due to multiple effects. With the increasing number of cells, all metabolites are produced in higher concentrations and might be pushed over the intensity threshold of the machine. Further, suboptimal growth conditions generally favour the production of diverse (secondary) metabolites^30^. At the same time, the number of measured molecules sorted as noise, decreases because low-intensity background features are suppressed by the increased overall intensity of biogenic molecules (Supplementary Fig. 2.1).

The shake flask experiment was designed to assess the reproducibility between technical as well as biological triplicates. As a threshold for reproducibility, we accepted an identified molecule if it is present in all three triplicate runs. Overall, between 50 and 75% of the identified features were present in technical replicates (Fig. 2a). The reproducibility increases over time with the number of identifiable features. Unsurprisingly, the overlap of measured features between the biological replicates is slightly smaller at 30 to 50% (Supplementary Fig. 2.2).

Although this is an acceptable number, the question arises, why not all measured features are present in all replicate runs. It is commonly postulated that this is due to currents of low-intensity molecules randomly sorted as biogenic. To take a closer look at this, the intensity for all molecules was determined, and the features were grouped depending on their appearance in one, two, or all three triplicate runs. The mean intensity of the molecules measured in all runs is distinctly higher than those present in just one or two runs (Fig. 2c and Supplementary Fig. 2.3). Taking an even closer look at the biological sample A at 6 h, the intensity for every measured molecule is shown on a logarithmic scale (Fig. 2d). Again, the mean and median intensity for those features measured in all three runs is higher. At the same time, it becomes evident that this difference is emphasized by a few particularly intense molecules and not by a sediment of non-reproducible low-intensity molecules.

A majority of the non-reproducible molecules might be an artefact of the insufficient identification procedure. Even deviations between measurements far smaller than 3 ppm can cause one of the triplicate features to be identified with multiple or non-matching molecular formulas, thus making this group no longer consistent. A visual representation is shown in Supplementary Fig. 2.4a. For example, in shake flask A at 6 h, in each of the triplicates a feature with [M + H]^+^ *m/z* 220.0929, 220.0930, or 220.0933 was measured (3 ppm = ± 0.0007 *m/z*). All three match the METLIN database entry C_7_H_13_N_3_O_5_, speculatively the asparagine-serine dipeptide, with the monoisotopic mass of [M + H]^+^ 220.0928. The last one, although being in the 3 ppm uncertainty range of C_7_H_13_N_3_O_5_, could also be C_8_H_17_N_3_S_2_ (*m/z* 220.0937). Considering features that could not be identified with a single fitting molecular formula lying within 3 ppm of identified features, up to 20 percentage points more features are present in all three triplicate runs (Supplementary Fig. 2.4b, c).

To connect the metabolites identified by the molecular formula to their biological function, the measured volatiles can be compared to the Yeast8 genome-scale model^10^, covering about 2100 entries for metabolites and hence the majority of the intrinsic yeast metabolism. In the measurement range of the SESI-Orbitrap of 50 to 500 Da lie just under 1000 molecules with in total 600 different molecular formulae, of which many are not volatile. Comparing this to the 200 tentatively identified volatiles in one experiment under one growth condition, it becomes apparent how much of the volatile space is jet to be explored. While none of the features measured in the uninoculated medium overlap with the Yeast8 model, nine molecules measured and identified during yeast growth overlap with metabolites in the model (Table 1). As the SESI was operated only in positive ionisation mode, metabolites such as acetate, which would be likely found in the negative ionisation mode, were not measured.

**Table 1:** Measured volatile metabolites that are also listed in the Yeast8 genome-scale model.

[M + H]^+^	Identification	Present ﻿at	Name (possible cell compartments)	
93.0911	Ethanol (dimer)	2 h, 4 h, 6 h	Ethanol (c, e, m)	
65.0597	Methanol (dimer)	4 h, 6 h	Methanol (c, e)	
75.0804	C_4_H_10_O	4 h, 6 h	Isobutanol (c, e, m)	
107.0525	C_4_H_10_OS	2 h, 4 h, 6 h	Methionol (c, e, m)	
89.0597	C_4_H_8_O_2_	4 h, 6 h	Acetaldehyde (c, e, m)	
89.0961	C_5_H_12_O	4 h, 6 h	2-Methylbutanol (c, e, m)	Isoamylol (c, e, m)
145.1223	C_8_H_16_O_2_	4 h, 6 h	Ethyl hexanoate (c, e, m)	Hexyl ethanoate (c, e, m)
173.1536	C_10_H_20_O_2_	4 h, 6 h	Ethyl octanoate (c, e, m)	
201.1849	C_12_H_24_O_2_	4 h, 6 h	Ethyl decanoate (c, e, m)	

Abbreviations for cell compartments: c (cytoplasm), e (extracellular), m (mitochondrion), n (nucleus).

As described above, ethanol can be identified conclusively. The same holds true for methanol (CH_4_O, dimer [M + H]^+^ 65.0597). In many applications, such as winemaking, the formation of methanol is regarded as the result of incomplete pectin degradation^31^. However, there are several other reactions in yeast that yield methanol as a by-product (Supplementary 2.4). For acetaldehyde (C_2_H_4_O), the dimer has the same molecular formula as acetoin or ethyl acetate (C_4_H_8_O_2_, [M + H]^+^ 89.0597), which were not detected using GC-FID or HPLC–UV/RI (data not shown). For 2 of the 7 remaining entries, two metabolites are possible.

### Online off-gas volatilome analytics using SESI-Orbitrap MS

**Figure 3:**
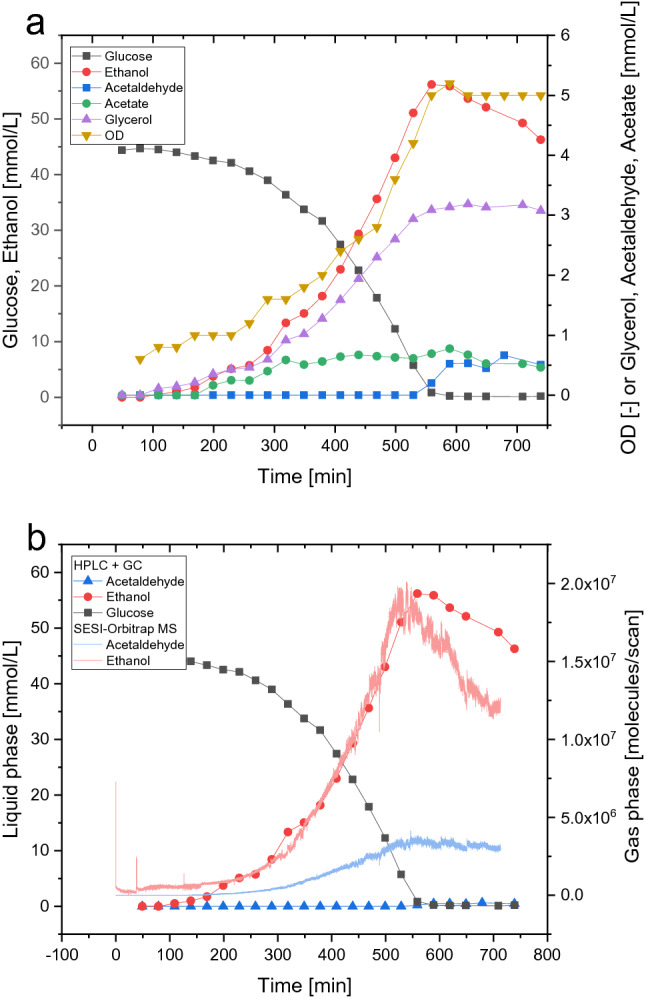
Results of the 200 mL scale yeast fermentation with online SESI-Orbitrap MS-based off-gas analytics. Optical density and common metabolites (measured by HPLC and GC) (**a**), agreement of the ethanol and acetaldehyde concentrations measured by HPLC and GC or SESI-Orbitrap MS (**b**).

While directly measuring shake flasks without the need for gas-phase sample preparation decreases the risk of generating artefacts, the true potential of the SESI-Orbitrap MS systems lies in online and real time off-gas analytics on a small-scale bioreactor. A double-walled glass reactor with a working volume of 200 mL was used. The empty, sterile reactor was filled with Verduyn minimal medium and equilibrated overnight at cultivation conditions: 30 °C, stirring at 800 rpm and gassing with compressed air at 400 m/L min (1.5 vvm) through a 0.8 mm needle. The next morning, the reactor was connected to the SESI-Orbitrap MS, and 30 min of the sterile reactor were measured as background (setup Supplementary Fig. 1.1) before the reactor was inoculated with washed *S. cerevisiae* S288C cells from a YEP preculture to an OD of 0.6. While the volatilome analysis was performed continuously online with over 16,000 measurements, HPLC, GC, and OD samples were taken every 30 min over 11.5 h. While glucose, ethanol, glycerol, and acetate were measured by HPLC–UV/RI, acetaldehyde was measured with the help of a GC-FID. The experiment was designed in such a way that two classic metabolic shifts could be observed. The first shift occurs between the lag- and growth phase. The lag phase was purposefully induced by using a complex medium preculture and minimal medium main culture. The second metabolic shift reflects the switching of the main C-source. Using glucose in concentrations above the Crabtree threshold (< 0.1 g depending on the strain used^28^), ethanol is produced during aerobic growth. Upon glucose depletion, the yeast can use ethanol via the conversion to acetaldehyde and acetate, and the subsequent anaplerotic reactions of the glyoxylate shunt into the TCA cycle to gain energy and support cell growth. After a lag phase of approximately 90–120 min, the yeasts grew until glucose depletion to an OD of 5 (Fig. 3a).

As already explained for the shake flask experiments, the measurement of ethanol production is of huge interest during yeast fermentations. Figure 3b shows the concentrations of ethanol and its metabolic precursor acetaldehyde as measured by HPLC and GC or the SESI-Orbitrap MS. As already shown for the shake flask experiments, the ethanol signal in the liquid- and gasphase are in good agreement. Ethanol was already detectable in the first taken HPLC sample after 30 min and instantly after inoculation in the SESI-Orbitrap MS. The first two visible spikes result from the pressure change upon the connection of the reactor to the machine and the inoculation. Interestingly, despite the coinciding ethanol signal for both methods during the first 500 min of the fermentation, the ethanol signal in the gas phase declines more rapidly after glucose depletion. This effect can be accounted to changes in the matrix: as described later, highly ionisable medium-chain fatty acid ethyl esters are produced in this phase which compete for ionisation in the SESI, thus quenching the ethanol signal. In the liquid samples analysed by GC-FID, acetaldehyde was visible only after glucose depletion at around 500 min. Contrasting that, the acetaldehyde signal in the SESI-Orbitrap MS rose above the background level at 120 min. Thus acetaldehyde, both as an interesting biotech product^32^ and an example of low-abundance high-volatile compounds was measured 380 min earlier, which could be of major interest for fermentation reaction control.

**Figure 4:**
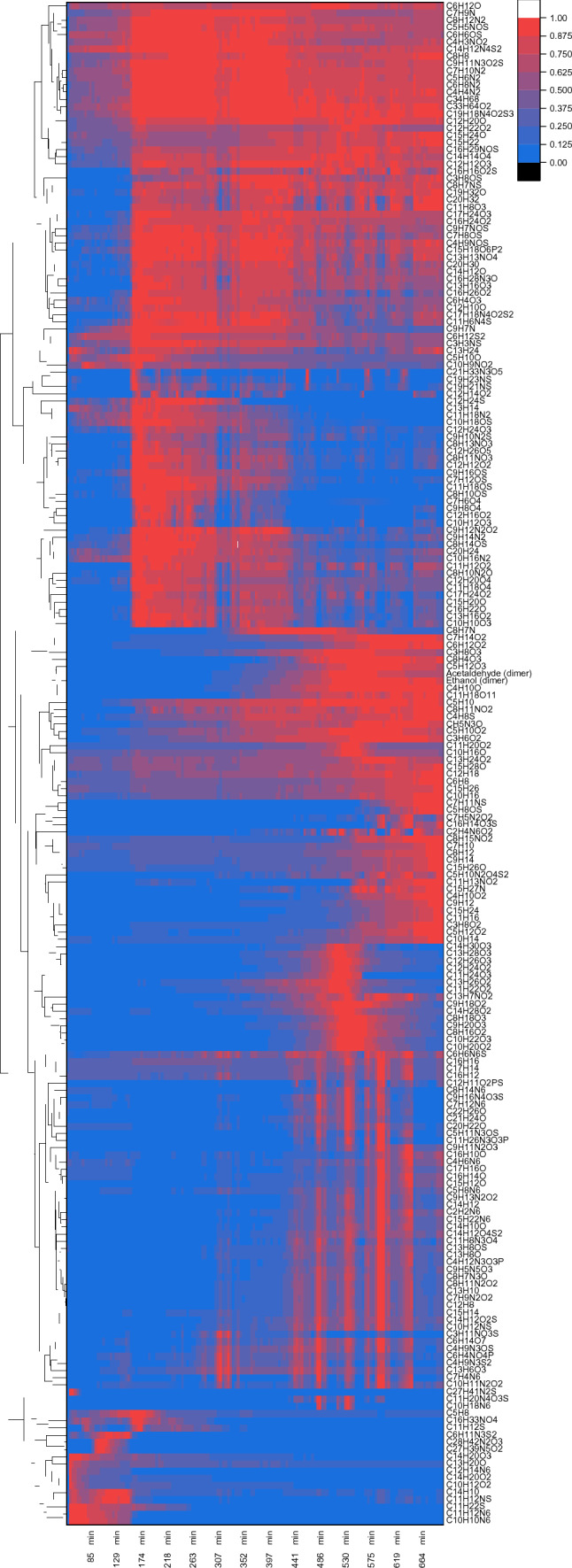
Results of the 200 mL scale yeast fermentation with online SESI-Orbitrap MS-based off-gas analytics. Heatmap of all tentatively identified metabolites measured throughout the experiment, clustered, and normalised to their individual maximum (blue to red, low to high intensity), every pixel shown is the mean of 100 scans, the first pixel represents the mean of the 30 min background.

Throughout the 11.5 h fermentation, over 2600 features were measured, of which close to 500 were sorted as biogenic. Molecules were defined to be of biogenic origin if their intensity increased at least three-fold compared to their background. Through comparison to the METLIN database^33^, 212 features were tentatively identified with a unique molecular formula. As described above, ethanol is uniquely identifiable and detectable throughout the whole fermentation. All molecules found in the fermentation off-gas are presented with their intensity profile in a clustered heatmap (Fig. 4). Because the measured intensities range over multiple orders of magnitude, all features were normalised to their own maximum set as 1. Again, the tentatively identified metabolites were matched against the Yeast8 genome-scale model^10^, yielding an overlap of eighteen substances, excluding methanol (Table 2). Six of these were also found in the shake flask experiments. To further strengthen their identification and showcase the possibilities of this system, the intensity of the isotope peak for these compounds was analysed (Supplementary Fig. 2.5). Isotopes occur naturally for most elements so that 1.08–1.10% of all carbon atoms are ^13^C instead of ^12^C. Thus, not only ethanol as ^12^C_2_H_6_O but also ^12^C^13^CH_6_O is present. The expected intensity of this species is the natural occurrence of the isotope times the number of respective atoms in the molecule, hence 2 × 1.08% = 2.16% of the mother peak. The mass resolution of the used SESI-Orbitrap MS allows for discrimination, for example, between the mass difference caused by the incorporation of a ^2^H against a ^13^C atom and the sensitivity is high enough to find the isotope peaks for most measured molecules.

**Table 2:** Measured volatile metabolites that are also listed in the Yeast8 genome-scale model.

[M + H]^+^	Identification	Name (possible cell compartments)	
**Also found in shake flask experiment**			
93.0911	Ethanol (dimer)	Ethanol (c, e, m)	
75.0804	C_4_H_10_O	Isobutanol (c, e, m)	
89.0597	C_4_H_8_O_2_	Acetaldehyde (c, e, m)	
145.1223	C_8_H_16_O_2_	Ethyl hexanoate (c, e, m)	Hexyl ethanoate (c, e, m)
173.1536	C_10_H_20_O_2_	Ethyl octanoate (c, e, m)	
201.1849	C_12_H_24_O_2_	Ethyl decanoate (c, e, m)	
**Not found in shake flask experiment**			
75.0441	C_3_H_6_O_2_	Propionate (c, p)	(S)-lactaldehyde (c)
93.0546	C_3_H_8_O_3_	Glycerol (c, e, lp)	
91.0754	C_4_H_10_O_2_	(R,R)-2,3-butanediol (c, e)	
87.0804	C_5_H_10_O	2-deoxy-D-ribose (c)	2-methylbutanal (c, e, m)
103.0754	C_5_H_10_O_2_	Ethyl propionate (c, e, m)	Propyl acetate (c, e)
117.0910	C_6_H_12_O_2_	Ethyl butanoate (c, e, m)	Isobutyl acetate (c, e)
131.1066	C_7_H_14_O_2_	Isoamyl acetate (c, e)	2-methylbutyl acetate (c, e)
170.0812	C_8_H_11_NO_3_	Pyridoxine (c, e)	
165.0910	C_10_H_12_O_2_	Phenethyl acetate (c, e)	
181.0859	C_10_H_12_O_3_	Tyrosyl acetate (c, e)	
176.0706	C_10_H_9_NO_2_	5-hydroxyindoleacetaldehyde (c)	
221.1900	C_15_H_24_O	2-trans,6-trans-Farnesal (c)	

Abbreviations for cell compartments: c (cytoplasm), e (extracellular), m (mitochondrion), n (nucleus), lp (lipid particle).

Each of the measured metabolites has a unique intensity curve. As with all online gas measurements, this system is susceptible to changes in the gas flow. The features between C_8_H_14_N_6_ and C_10_H_11_N_2_O_2_ in the lower half of the heatmap are particularly striking because of their ribbon pattern with half-hourly repetition. These coincide with the HPLC and GC sampling and changes in the total ion current (TIC, Supplementary Fig. 2.7). Therefore, this can be accounted to an altered gas flow. Nevertheless, most of them are still much more dominant in the later fermentation phase, and the sampling-derived artefacts could be interpolated. In addition to these molecules, three groups of metabolites are distinguishable: metabolites that show a distinct increase after the lag phase, those increasing in intensity throughout the fermentation, and those which are just present during the C-source shift (Supplementary Fig. 2.6).

Of those metabolites that undergo a drastic change in intensity at the end of the lag phase, four are listed in the Yeast8 model. The lag phase is not the mundane “waiting-to-start” period as it is often perceived, but a dynamic period preparing microbes for cell division^34^. In baker’s yeast, intense gene regulation occurs and 240 open reading frames are at least five-fold induced, and 122 are at least fivefold repressed^35^. Two of the measured metabolites are connected to amino acid degradation pathways: C_5_H_10_O, presumably 2-methylbutanal, based on the low vapour pressure of 2-deoxy-D-ribose, is a known intermediate in L-isoleucine degradation; C_10_H_12_O_3_ tyrosyl acetate is produced via acetylation of tyrosol, an end-product of tyrosine degradation. It is known that the intracellular free amino acid concentration is highest during the lag-phase^36^. Therefore, the advent of amino acid breakdown products is expected upon entering the growth phase. Further, pyridoxine (C_8_H_11_NO_3_) is a precursor in the synthesis of pyridoxal phosphate (PLP), the active form of vitamin B6, which is a cofactor in a plethora of different reactions, and 5-hydroxyindoleacetaldehyde (C_10_H_9_NO_2_) is a breakdown product of the yeast metabolite serotonin^37^.

From those molecules just present during the C-source shift, three are listed in the Yeast8 model. All of them are medium-chain fatty acid (MFA) ethyl esters (ethyl hexanoate, ethyl octanoate, ethyl decanoate), whose biological functions are only sparsely explored^38^. During glucose-supported growth, fatty acids are synthesised, but this stops upon glucose depletion. Therefore MFA ethyl ester formation is, in this case, most likely a mechanism to free CoA bound to MFA and, at the same time, detoxify free fatty acids, as they disturb the cellular pH^38^.

In this work, we explored the application of SESI-Orbitrap MS to measure the yeast volatilome in quick and easy measurements over shake flasks or over a prolonged fermentation time from the off-gas. On the example of *S. cerevisiae*’s prime metabolite ethanol, we show the quantitative agreement between fermentation broth analytics using HPLC–UV/RI and gas-phase SESI-Orbitrap MS measurements. Further, we showcase on the example of acetaldehyde that this system offers new possibilities to track in real-time low-abundance, high-volatility metabolic intermediates. On the example of a yeast culture growing on minimal medium, the volatilome was measured over 16,000 times over 11.5 h. Two metabolic shifts were observable. At the end of the lag phase, primarily amino acid degradation products were observable. And upon the C-source shift, MFA ethyl esters were dominant. Overall, the usability of SESI-Orbitrap MS was shown for both cultivation monitoring and exploration of yeast secondary metabolism.

In combination with transcriptomic methods, SESI-Orbitrap MS could provide new insights into cellular stress responses and their regulation. For example, it could elucidate the role of MFA ethyl ester formation. Coupling this sensitive system with online capabilities to fermentation control equipment could improve fermentations, as the response time can be in seconds rather than minutes.

## Material and methods

### Yeast cultivation in shake flasks and 200 mL scale reactor

The haploid laboratory *S. cerevisiae* strain S288C was used for all experiments. Optical density at 600 nm (OD) was determined with an Ultrospec 10 (Amersham Biosciences, Little Chalfont, UK) photometer. All cultivations were performed at 30 °C and 300 rpm. For shake flask experiments, a preculture was grown in 100 mL Verduyn minimal medium with 10% glucose (a more detailed composition, Supplementary 1.1). On the day of the experiment, the preculture was diluted and split into three 100 mL shake flasks containing 25 mL each with an OD of 2.0.

For reactor experiments, a preculture was grown in 100 mL yeast extract peptone (YEP, a more detailed composition, Supplementary 1.1). The reactor and setup was used as described by Mengers et al.^32^. In short, a double-walled glass reactor with a working volume of 200 mL was heated to 30 °C with a thermostat, stirred at 800 rpm with a triangular, 3.7 cm long stirring bar, and gassed with compressed, humidified air at a rate of 400 mL/min through a needle with 0.8 mm diameter (Supplementary Fig. 1.1).

### GC-FID and HPLC analytics

The GC-FID and HPLC–UV/RI methods were used as described by Mengers et al.^32^. In short, for GC-FID measurements, the samples were diluted 1:20 with acetonitrile and filtered with Berrytec CA 0.22 µm disposable syringe filters before dilution if the sample contained biomass. Samples were centrifuged before storage in GC vials. A Trace GC Ultra GC-FID (Thermo Scientific, Waltham, MA, USA) was used with a 30 m Zebron ZB-WAX column with an inner diameter of 0.25 mm. The optimized sequence parameters are the following: a sample volume of 0.1 mL, a flow rate of 1 mL/min with helium as carrier gas, and a split ratio of 1:10. The temperature program was as follows: 50 °C for 8 min, increased by 27.5 °C/min to 160 °C, and kept constant for 3 min. The inlet was kept at 250 °C.

For HPLC analytics, all samples containing biomass were filtered through Berrytec CA 0.22 µm disposable syringe filters before analysis. A DIONEX UltiMate 3000 HPLC System (Thermo Scientific, Waltham, MA, USA) with a Metab-AAC column (300 × 7.8 mm column, ISERA, Düren, Germany) was used. Elution was performed with 5 mM H_2_SO_4_ at a flow rate of 0.4 mL/min and a temperature of 40 °C. For detection, a SHODEX RI-101 detector (Showa Denko Europe GmbH, München, Germany) and a DIONEX UltiMate 3000 Variable Wavelength Detector set to 210 nm were used. The glucose peaks were normalised to the phthalate peaks.

### SESI-Orbitrap mass spectrometry

A Super-SESI unit (Fossiliontech, Madrid, Spain) was coupled to a Q Exactive Orbitrap mass spectrometer (Thermo Fisher Scientific, Bremen, Germany).Mechanically sharpened nano-electrospray emitters Sharp Singularity (Fossiliontech) were used for all measurements. The SESI intake line was heated to 100 °C, the SESI core to 130 °C, and the intake capillary to 320 °C. The method described by Mengers et al. (currently under review) was used. The scan range was set to 50–500 *m/z* with a resolution of 70,000 and with 10 microscans, resulting in a speed of approximately 0.4 Hz. (For more details, see S1.2). The lock masses used are depicted in Table S1.2. External mass calibration was performed regularly, and the mass shift of the machine was usually between 2 and 3 ppm (Supplementary Table 2.4).

For the measurement of the yeast volatilome in shake flasks, the SESI-Orbitrap MS was set in suction mode by setting the auxiliary gas to 0 a. u., which resulted in an air intake of approximately 800 mL/min. A background of laboratory air was measured for 1 min before the shake flask was put directly under the extended intake line (For a picture of the setup, see Fig. S1.1) for 2 min. For the online measurement of yeast fermentation off-gas, the auxiliary gas was set to 2 a.u. The reactor was aerated with 400 mL/min (2 vvm), and the airflow was split with a glass 3-neck olive with 300 mL/min into the SESI and 100 mL/min to the overflow. This was done to condensate off-gas moisture partially. (For a picture of the setup, see Fig. S1.1). Here a background of 30 min of the inoculated medium was measured as a reference.

### Data treatment and analysis

The data treatment follows the same protocol as described by Mengers et al. (currently under review). In short, the raw files were converted to Excel format with the intensity over time profile for every feature using the open-source software MZmine2^39^. While doing this, the following data processing steps were applied: scan-to-scan filtering with a Savitzky-Golay filter (5 data points), mass detection (noise cut-off 1E04), and ADAP-chromatogram builder (minimum group size 30 scans, minimum intensity 1E04). The Excel files were post-processed with a self-written Python script, which sorted the measured features in *noise* and *sample derived* based on their relative intensity compared to the background. Compound identification was performed by matching *m/z* values against the METLIN database^33^.

## Supplementary Information

Supplementary Information.

Supplementary Figures.

## Data Availability

Materials and data are made available on request.
