# Supplementary material for: Using off-gas for insights through online monitoring of ethanol and baker’s yeast volatilome using SESI-Orbitrap MS
Source: Sci Rep. 2022 Jul 21;12:12462. doi: 10.1038/s41598-022-16554-z (PMC9304407; doi:10.1038/s41598-022-16554-z)
Supplement: Supplementary file 1 — Supplementary Information. [file 41598_2022_16554_MOESM1_ESM.docx]

Using off-gas for insights through online monitoring of ethanol and baker’s yeast volatilome using SESI-Orbitrap MS

Hendrik G. Mengers^a^, Martin Zimmermann^a^, Lars M. Blank^a^

HGM https://orcid.org/0000-0002-5098-164X, LMB https://orcid.org/0000-0003-0961-4976

a. Institute of Applied Microbiology - iAMB, Aachener Biology and Biotechnology - ABBt, RWTH Aachen University, Aachen, Germany

**Corresponding author:** Lars M. Blank lars.blank@rwth-aachen.de

**Table of contents**

[1. Supplementary Material and Methods 2](#_Toc102030508)

[1.1 Yeast cultivation 2](#_Toc102030509)

[1.2 SESI-Orbitrap mass spectrometry 2](#_Toc102030510)

[1.3 Setup for yeast volatilome measurements 3](#_Toc102030511)

[1.4 Data treatment and compound identification 3](#_Toc102030512)

[2. Supplementary Results 4](#_Toc102030513)

[2.1 Yeast volatilome in shake flasks 4](#_Toc102030514)

[2.2 Mean intensity of features present in 1, 2, or 3 triplicate runs 5](#_Toc102030515)

[2.3 Non-consistent triplicate measurements because of insufficient identification 5](#_Toc102030516)

[2.4 Yeast reactions yielding methanol 7](#_Toc102030517)

[2.5 Analysis of isotope peaks for the volatilome measurement from fermentation off-gas 7](#_Toc102030518)

[2.6 Metabolites present during metabolic shifts 10](#_Toc102030519)

[2.7 Assessment of the SESI-Orbitrap MS measurement mass shift 10](#_Toc102030520)

[References 12](#_Toc102030521)

# Supplementary Material and Methods

## 1.1 Yeast cultivation

Verduyn minimal medium with 0.75 % glucose contained 7.5 g/L glucose, 10 g/L potassium hydrogen phthalate, 2.3 g/L urea, 3 g/L, KH_2_PO_4_, 0.5 g/L MgSO_4_ 7 H_2_O, as well as 10 mL/L 100x trace elements, 1 mL/L of 1000x vitamins. The vitamin solution contained 0.05 g/L D-biotin, 1 g/L calcium D pantothenate, 1 g/L nicotinic acid, 25 g/L myo-inositol, 1 g/L thiamine hydrochloride, 1 g/L pyridoxine hydrochloride and 0.2 g/L p-aminobenzoic acid. The trace element solution consisted of 1.5 g/L EDTA, 0.45 g/L ZnSO_4_·7 H_2_O, 0.1 g/L MnCl_2_·4 H_2_O, 0.03 g/L CoCl_2_·7 H_2_O, 0.03 g/L CuSO_4_·5 H_2_O, 0.04 g/L NaMoO_4_·2 H_2_O, 0.45 g/L CaCl_2_·2 H_2_O, 0.3 g/L FeSO_4_·7 H_2_O, 0.1 g/L H_3_BO_3_ and 0.01 g/L KI. The pH was adjusted to 5 with KOH before sterile filtration.

Precultures for the 200 mL scale fermentation were performed in shake flasks with YEP medium containing 10 g/L yeast extract, 20 g/L peptone, and 20 g/L glucose. The flasks were inoculated with 10 µl of a frozen stock and cultivated at 30 °C for 48 h. Before inoculation, the cells were centrifuged for 3 min at 3,500 x g and subsequently washed with Verduyn minimal medium.

## 1.2 SESI-Orbitrap mass spectrometry

Table 1.1: Settings used for the experiments, if not otherwise mentioned

| **Section** | **Property** | **Value** |
| --- | --- | --- |
| Method | Resolution | 70,000 |
|  | AGC target | 1E06 |
|  | Maximum IT | Auto |
|  | Spectrum data type | Profile |
| Tune file | Sheath gas flow rate | 10 a. u. |
|  | Aux gas flow rate | Shake flask experiments: 0 a. u.  Reactor off-gas measurement: 2 a.u. |
|  | Spray voltage | 2.5 kV |
|  | Capillary temperature | 320 °C |
|  | S-lens RF level | 50 |
| SESI | Intake line temperature | 100 °C |
|  | Ionizer core temperature | 130 °C |

Table 1.2: Lock masses used for the experiments

| **MS Polarity** | **Exact Adduct Mass** | **Compound** | **Formula (excluding ionization)** |
| --- | --- | --- | --- |
| Positive | 149.0233 | phthalic anhydrate | C_8_H_4_O_3_ |
| Positive | 279.1591 | diisobutyl phthalate | C_16_H_22_O_4_ |
| Positive | 445.1200 | polysiloxane (n6) | C_24_H_36_Si_6_O_6_ |
| Positive | 158.96403 | sodium trifluoroacetate | C_2_F_3_NaO_2_ |
| Positive | 107.07027 | polypropylene glycole | C_3_H_6_O |
| Positive | 214.08963 | n-butyl benzenesulfonamide (plasticizer) | C_10_H_15_NO_2_S |

## 1.3 Setup for yeast volatilome measurements


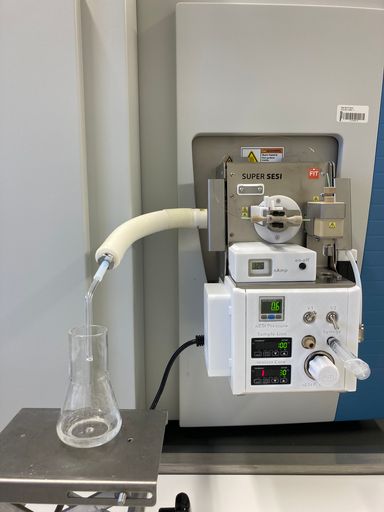

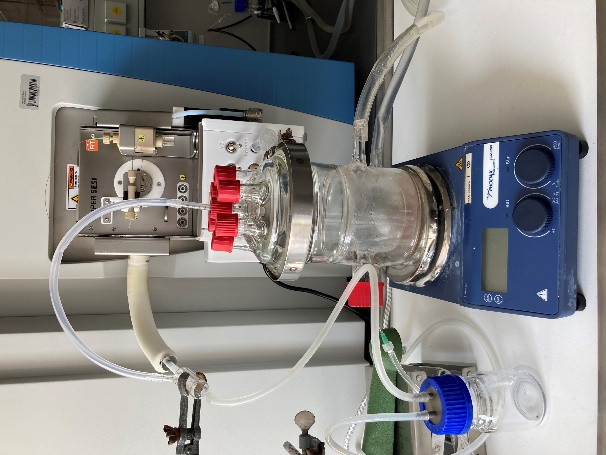


Figure 1.1: The setup for the measurement of shake-flasks (left) and online measurement of fermentation off-gas (right)

## 1.4 Data treatment and compound identification

For matching the measured m/z values with four decimal places against the METLIN database^1^, the following settings were used: Tolerance of 3 ppm, charge positive, Adduct M+H, add peptides, toxicants, and KEGG IDs to search. Results containing one of the following were not considered as possible identifications: halogens, metals, phosphorus (if less than 2 oxygen per phosphorous), salts, and combination of molecules.

# Supplementary Results

## 2.1 Yeast volatilome in shake flasks

| a | b |
| --- | --- |

Figure 2.1: Sorting of features measured during shake flask experiments into noise and biogenic (a) and the number of features identified (b).

|  |
| --- |

Figure 2.2: Percentage of features, that are present in all replicates over time between technical replicates (grey, red, blue) and between the biological replicates (green), here just features present in all technical replicates were used.

## 2.2 Mean intensity of features present in 1, 2, or 3 triplicate runs

| a | b |
| --- | --- |
| c |  |

Figure 2.3: Mean intensity of features present in one, two or all three triplicate runs, error bars are just presented for n=3. Fig. 2-3a is also depicted in the main manuscript.

## 2.3 Non-consistent triplicate measurements because of insufficient identification

As described above, identification was performed by comparing the m/z values against the METLIN database with a ± 3 ppm uncertainty. Here a feature could match no molecular formula (no identification), exactly one molecular formula, or multiple molecular formulae. Supplementary Fig. 2.4a shows how three features lying within a 3 ppm radius can be divergently identified. M1 and M2 are solely matched with database entry 1, while M3 also fits both database entry 1 and entry 2. This means, that these measurements would appear non-reproducible, as one has a different identification (multiple instead of entry 1). To check whether the insufficient identification causes consistent triplicate results to appear as non-consistent, the data was checked if a feature with “no identification” or “multiple identifications” lies within 3 ppm of the identified features (Supplementary Fig. 2.4b). This changes up to 20 percentage points of features to be reproducible (Supplementary Fig. 2.4c). But this procedure was not used to enhance the data for the volatilome analysis.

| a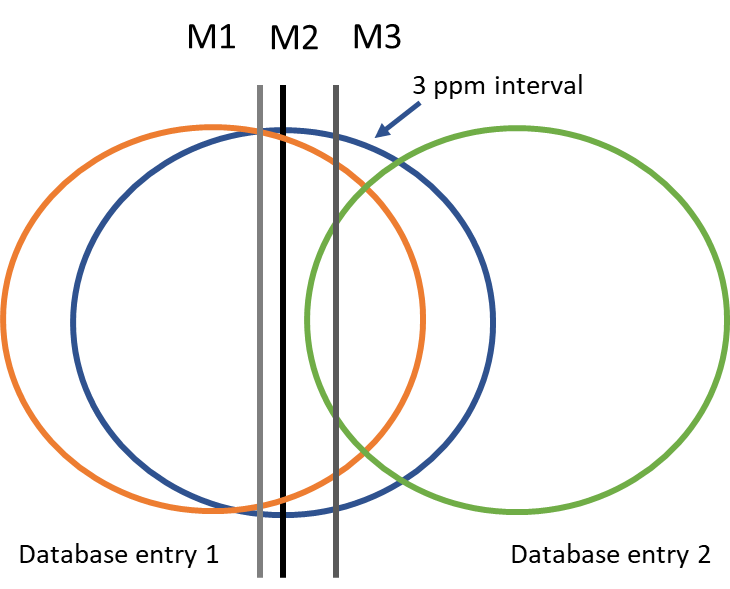 | b |
| --- | --- |
| c  |  |

Figure 2.4: Visual representation of how insufficient identification can cause non-consistent triplicate experiments (a). Percentage of features that are present in 1, 2, or three runs with the addition of non-identified features within 3 ppm of the identified features (b) and the percentage change this makes for the reproducibility (c)

## 2.4 Yeast reactions yielding methanol

The YeastPathways database shows reactions and pathways for *S. cerevisiae* based on the entries of the *Saccharomyces* Genome Database (SGD)^2^ and the software Pathway tools^3^. All six reactions known to yield methanol are shown in Tab. 2.1.

Table 2.1: Reactions and enzymes in S. cerevisiae known to yield methanol as a by-product

| **EC number** | **Enzyme** | **Educts** | **Products** |
| --- | --- | --- | --- |
| 3.1.1.61 | carboxyl methyl esterase: Ppe1 | a [protein]-L-glutamate-O5-methyl-ester + H_2_O | a [protein]-α-L-glutamate + methanol + H^+^ |
| 1.1.1.2 | NADPH-dependent alcohol dehydrogenase: Adh6 | an aldehyde + NADPH + H^+^ | an alcohol + NADP^+^ |
| 3.1.3.2 | acid phosphatase: Pho12, Pho3, Pho5, Pho11 | a phosphate monoester + H_2_O | an alcohol + phosphate |
| 3.1.3.1 | alkaline phosphatase: Pho8 | a phosphate monoester + H_2_O | an alcohol + phosphate |
| 3.1.4.46 | --- | a glycerophosphodiester + H_2_O | an alcohol + *sn*-glycerol 3-phosphate + H+ |

## 2.5 Analysis of isotope peaks for the volatilome measurement from fermentation off-gas

As described above, the identification of the measured features was done by comparing the exact mass determined by high-resolution MS to the METLIN database. To strengthen the identification, one can analyse not only the exact mass of the mother peak but also the relative intensity of the compound's isotope peaks.

A list of the naturally occurring isotopes and their abundance, for the important elements C, H, N, O; and S is shown in Tab. 2.2. The mass spectrometer used in this study has a resolution of 70,000 and works with an accuracy of ± 3 ppm and is, therefore, able to resolve the differences of isotope peaks for example caused by ^13^C and ^2^H, as demonstrated by Tab 2.3.

Table 2.2: exact mass of the elements important to this study with their relative abundance ^4^

| **Element** | **Symbol** | **m** | **m+1** | | **m+2** | |
| --- | --- | --- | --- | --- | --- | --- |
| Carbon | C | 12.00000 | 1.08 % | 13.00335 | --- | --- |
| Hydrogen | H | 1.00782 | 0.115 % | 2.01410 | --- | --- |
| Nitrogen | N | 14.00307 | 0.368 % | 15.00010 | --- | --- |
| Oxygen | O | 15.99491 | 0.038 % | 16.99913 | 0.205 % | 17.99916 |
| Sulfur | S | 31.97207 | 0.76 % | 32.97145 | 4.29 % | 33.96786 |

Table 2.3: Mass shifts caused by isotopes and the resulting ppm shift assuming a molecule with 200 Da.

| **For M+1 with ^13^C** | | |
| --- | --- | --- |
|  | **Δ M** | **ppm at 200 Da** |
| ^13^C | 1.00335 | 0 |
| ^2^H | 1.00628 | 14.6 |
| ^15^N | 0.99703 | 31.4 |
| ^17^O | 1.00422 | 4.3 |
| ^33^S | 0.99938 | 19.8 |

The intensity of the isotope peak was analysed for all molecules that were measured and are also listed in the Yeast8 genome-scale metabolic model. The expected intensity of the ^13^C isotope peaks is calculated as the number of carbon atoms in the molecular formula multiplied by the natural abundance of the isotope, i.e. 1.08 %. The ^13^C signal in relation to the mother peak throughout the fermentation is depicted in Supplementary Fig. 2.5. If the intensity of the mother peak is zero, no value for the relative intensity is given. If the intensity of the isotope peak is zero, the relative intensity is also zero. The signal quality with the SESI-Orbitrap MS depends partly on the intensity. As the isotope peaks have an intensity of 5-10 % of the mother peak, some of them are at the lower detection range. Most likely because of the low intensity, no isotope peak was found for the following molecules: C_3_H_8_O_3_ (glycerol), C_3_H_8_O_3_ ((R,R)-2,3-butanediol), C_5_H_10_O_2_ (ethyl propionate)_,_ C_8_H_11_NO_3_ (pyridoxine), and C_10_H_12_O_3_ (Tyrosyl acetate). Overall, the measured and expected intensity coincides for all isotope peaks. For many compounds, the smoothed signal shows an increase of the relative isotope signal at the beginning, which is an artefact. Upon production of these metabolites, they are present at very low concentrations, which is expressed by the comparatively high fluctuations in the signals. In many of the individual scans, these compounds are below the threshold and have therefore an intensity of zero. The same is true for phenethyl acetate in the period between 400-600 minutes and 5-hydroxyindole acetaldehyde during the whole experiment.

Figure 2.5: Relative intensity of the ^13^C isotope peak in relation to the mother peak. For clarity, additionally smoothed data is shown (Savitzky-Golay filter with 50 data points). A line indicates the expected intensity based on the molecular formula and natural abundance of ^13^C.

## 2.6 Metabolites present during metabolic shifts

Both the data from acetate and ethanol as measured by HPLC, as well as the parts of the heatmap presented here, are also depicted in the main paper.


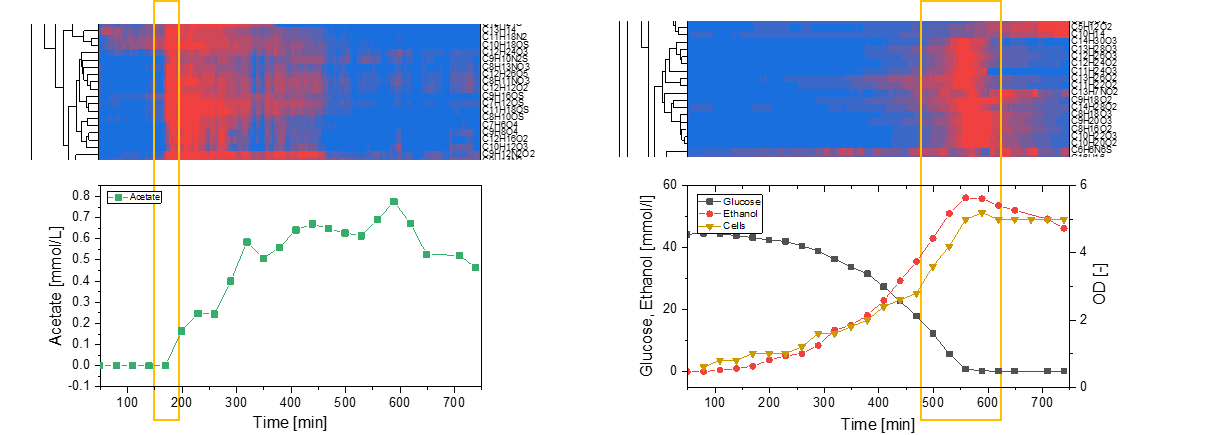


Figure 2.6: Timely correlation of metabolite concentrations measured by HPLC and SESI-Orbitrap MS. The heatmaps shown are fragments of the one presented in the main paper in Fig. 4

## 2.7 Assessment of the SESI-Orbitrap MS measurement mass shift

As with every measuring device, the SESI-Orbitrap MS has a level of inaccuracy, usually given as shift in parts per million. At an m/z with [M+H]^+^ of 100.0000 a shift of ± 3 ppm would mean an inaccuracy range of m/z 99.9997 – 100.0003. The mass shift of each identified feature is determined by the mass difference to the theoretical [M+H]^+^. For the determination of the mean shift, the absolute value of each mass shift was used.

Table 2.4: Mean mass shift of the identified molecules for the performed experiments with the respective standard deviation.

| **Measurement** | **Biological Replicate** | **Tech. replicate** | **Mean shift** | **Standard deviation** |
| --- | --- | --- | --- | --- |
| Uninoculated medium | Triplicate 1 | | 1.19 | 0.80 |
|  | Triplicate 2 | | 0.91 | 0.64 |
|  | Triplicate 3 | | 1.07 | 0.68 |
| 2 h | A | Triplicate 1 | 1.40 | 0.84 |
|  |  | Triplicate 2 | 1.21 | 0.79 |
|  |  | Triplicate 3 | 1.14 | 0.84 |
|  | B | Triplicate 1 | 1.35 | 0.90 |
|  |  | Triplicate 2 | 1.34 | 0.81 |
|  |  | Triplicate 3 | 1.33 | 0.82 |
|  | C | Triplicate 1 | 1.25 | 0.91 |
|  |  | Triplicate 2 | 1.28 | 0.89 |
|  |  | Triplicate 3 | 1.19 | 0.88 |
| 4 h | A | Triplicate 1 | 1.44 | 0.79 |
|  |  | Triplicate 2 | 1.33 | 0.85 |
|  |  | Triplicate 3 | 1.34 | 0.83 |
|  | B | Triplicate 1 | 1.35 | 0.83 |
|  |  | Triplicate 2 | 1.37 | 0.83 |
|  |  | Triplicate 3 | 1.39 | 0.84 |
|  | C | Triplicate 1 | 1.35 | 0.83 |
|  |  | Triplicate 2 | 1.28 | 0.82 |
|  |  | Triplicate 3 | 1.29 | 0.87 |
| 6 h | A | Triplicate 1 | 1.36 | 0.86 |
|  |  | Triplicate 2 | 1.41 | 0.82 |
|  |  | Triplicate 3 | 1.28 | 0.79 |
|  | B | Triplicate 1 | 1.38 | 0.82 |
|  |  | Triplicate 2 | 1.31 | 0.81 |
|  |  | Triplicate 3 | 1.35 | 0.79 |
|  | C | Triplicate 1 | 1.36 | 0.83 |
|  |  | Triplicate 2 | 1.33 | 0.83 |
|  |  | Triplicate 3 | 1.31 | 0.80 |

## 2.8 Total ion current during yeast fermentation

The total ion current (TIC) is the total number of ions per scan. A sudden change in the TIC can indicate for example altered gas flow.

Figure 2.7: Total ion current over time for the 200 mL scale fermentation.

References

1. Smith, C. A. *et al.* METLIN: a metabolite mass spectral database. *Ther. Drug. Monit.* **27,** 747–751; 10.1097/01.ftd.0000179845.53213.39 (2005).

2. Cherry, J. M. *et al. Saccharomyces* Genome Database: the genomics resource of budding yeast. *Nucleic Acids Res.* **40,** D700-5; 10.1093/nar/gkr1029 (2012).

3. Karp, P. D. *et al.* Pathway Tools version 24.0: integrated software for pathway/genome informatics and systems biology, 14.10.2015.

4. Berglund, M. & Wieser, M. E. Isotopic compositions of the elements 2009 (IUPAC Technical Report). *Pure Appl. Chem.* **83,** 397–410; 10.1351/PAC-REP-10-06-02 (2011).
